# Supplementary material for: Pentafluorophenyl Platinum(II) Complexes of PTA and Its N-Allyl and N-Benzyl Derivatives: Synthesis, Characterization and Biological Activity
Source: Materials (Basel). 2019 Nov 26;12(23):3907. doi: 10.3390/ma12233907 (PMC6926962; doi:10.3390/ma12233907)
Supplement: Supplementary file 1 [file materials-12-03907-s001.pdf]

materials

Electronic Supplementary Information (ESI)

Pentafluorophenyl Platinum(II) Complexes of PTA and its N-Allyl and N-Benzyl Derivatives: Synthesis, Characterization and Biological Activity

Paolo Sgarbossa [1,*], Urszula Śliwińska-Hill [2], M. Fátima C. Guedes da Silva [3], Barbara Bażanów [4], Aleksandra Pawlak [5], Natalia Jackulak [4], Dominik Poradowski [6], Armando J. L. Pombeiro [3] and Piotr Smoleński [7,*]

1. Dipartimento di Ingegneria Industriale and CIRCC, Consorzio Interuniversitario per le Reattività Chimiche e la Catalisi, Università di Padova, via Marzolo 9, 35131 Padova, Italy; paolo.sgarbossa@unipd.it
2. Department of Analytical Chemistry, Faculty of Pharmacy, Wrocław Medical University, Borowska 211 A, 50-566 Wrocław, Poland; urszula.sliwinska-hill@umed.wroc.pl
3. Centro de Química Estrutural, Instituto Superior Técnico, Universidade de Lisboa, Av. Rovisco Pais, 1049-001 Lisboa, Portugal; fatima.guedes@tecnico.ulisboa.pt (M.F.C.G.d.S.); pombeiro@tecnico.ulisboa.pt (A.J.L.P.)
4. Department of Veterinary Microbiology, Wrocław University of Environmental and Life Sciences, Norwida 31, 50-375 Wrocław, Poland; barbara.bazanow@upwr.edu.pl (B.B.); natalia.jackulak@upwr.edu.pl (N.J.)
5. Department of Biochemistry, Pharmacology and Toxicology, Wrocław University of Environmental and Life Sciences, Norwida 31, 50-375 Wrocław, Poland; aleksandra.pawlak@upwr.edu.pl
6. Department of Biostructure and Animal Physiology, Wrocław University of Environmental and Life Sciences, Kożuchowska 1/3, 51-631 Wrocław, Poland; dominik.poradowski@upwr.edu.pl
7. Faculty of Chemistry, University of Wroclaw, F. Joliot-Curie 14, 50-383 Wrocław, Poland; piotr.smolenski@chem.uni.wroc.pl

* Correspondence: paolo.sgarbossa@unipd.it (P. Sgarbossa); piotr.smolenski@chem.uni.wroc.pl (P. Smoleński)

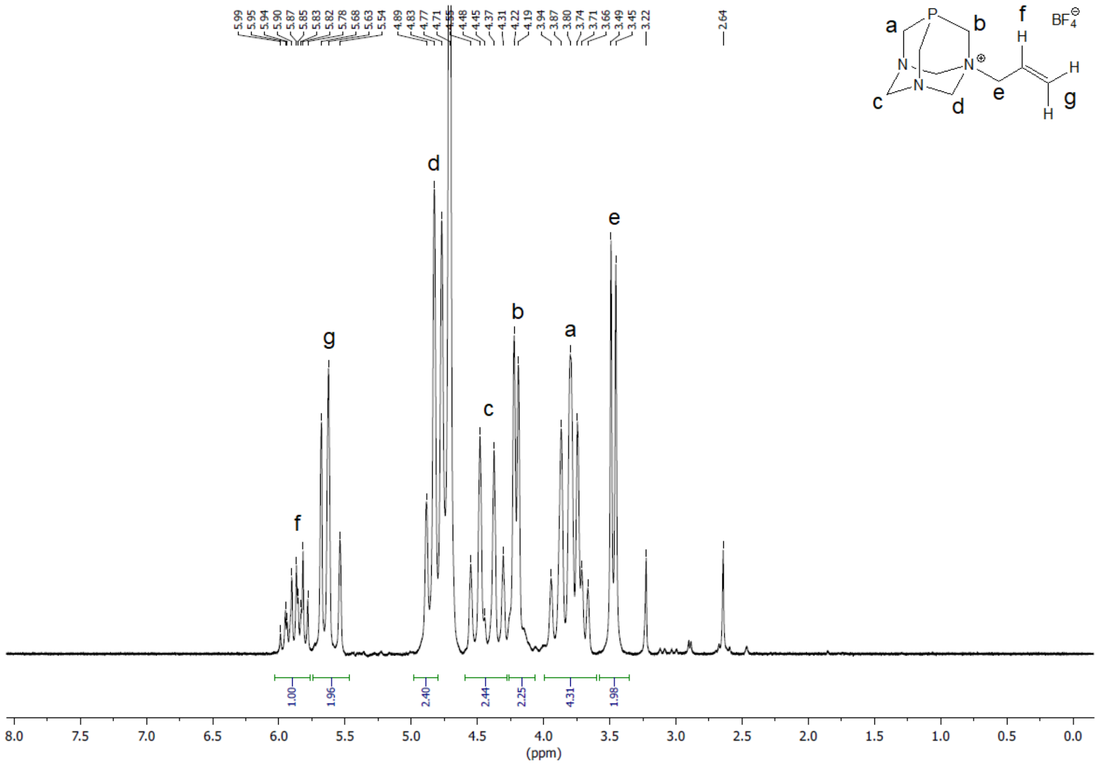

Figure S1. ^{1}H NMR spectrum of ligand **1b**.

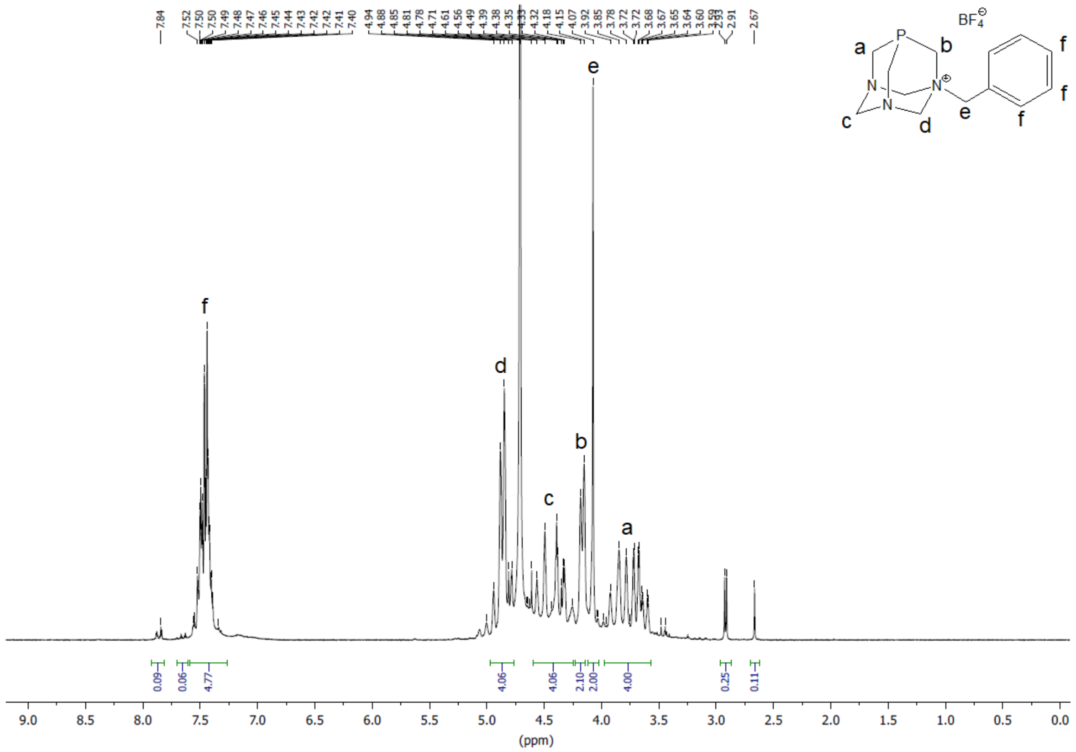

Figure S2. ^{1}H NMR spectrum of ligand **1c**.

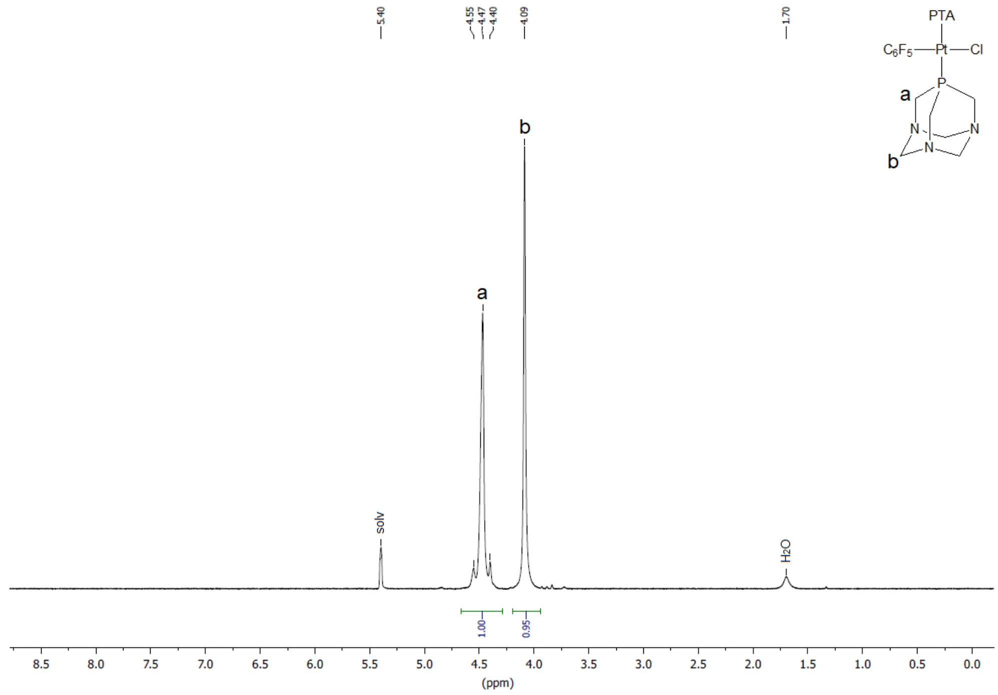

Figure S3. ^{1}H NMR spectrum of complex **2a**.

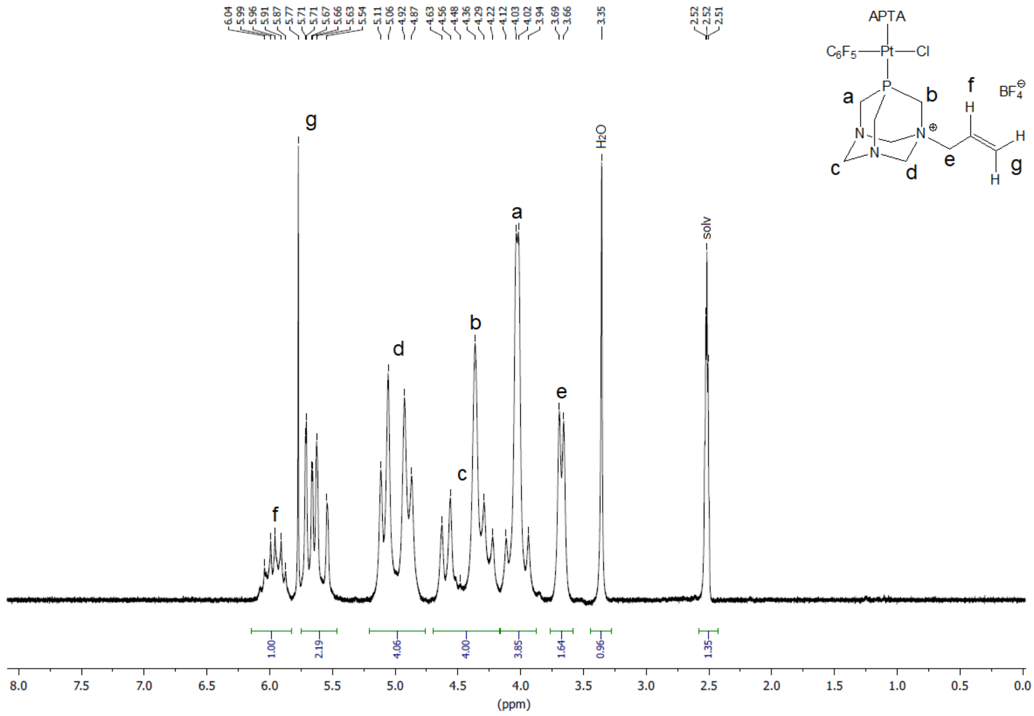

Figure S4. ^{1}H NMR spectrum of complex **2b**.

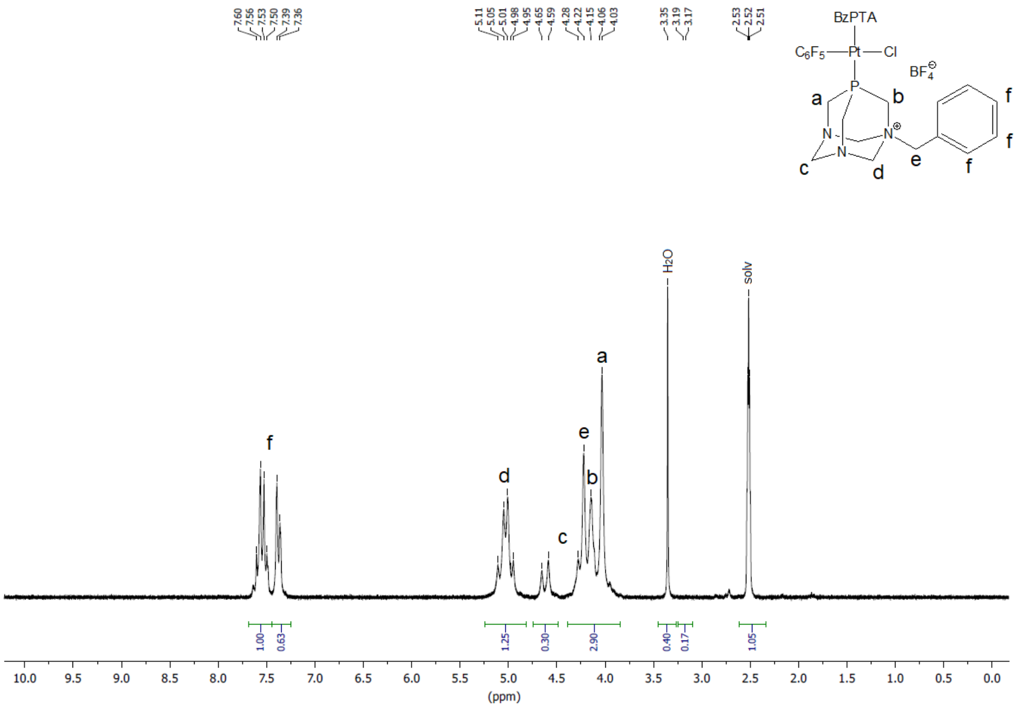

Figure S5. ^{1}H NMR spectrum of complex **2c**.

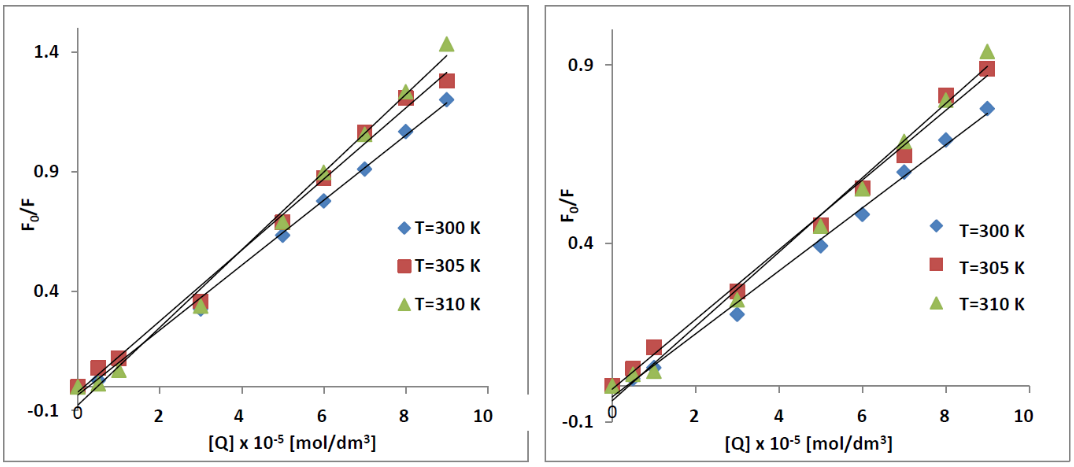

Figure S6. Stern-Volmer plots for quenching of HSA fluorescence by **2b** (left) and **2c** (right) at different concentrations and temperatures (300 K, 305 K, 310 K). [HSA] = 10 µM, [complex] = 5, 10, 30, 50, 60, 70, 80 and 90 µM; pH = 7.40, 0.05 M phosphate buffer, incubation at 37 °C during 24 h, λ_{ex} = 295 nm.

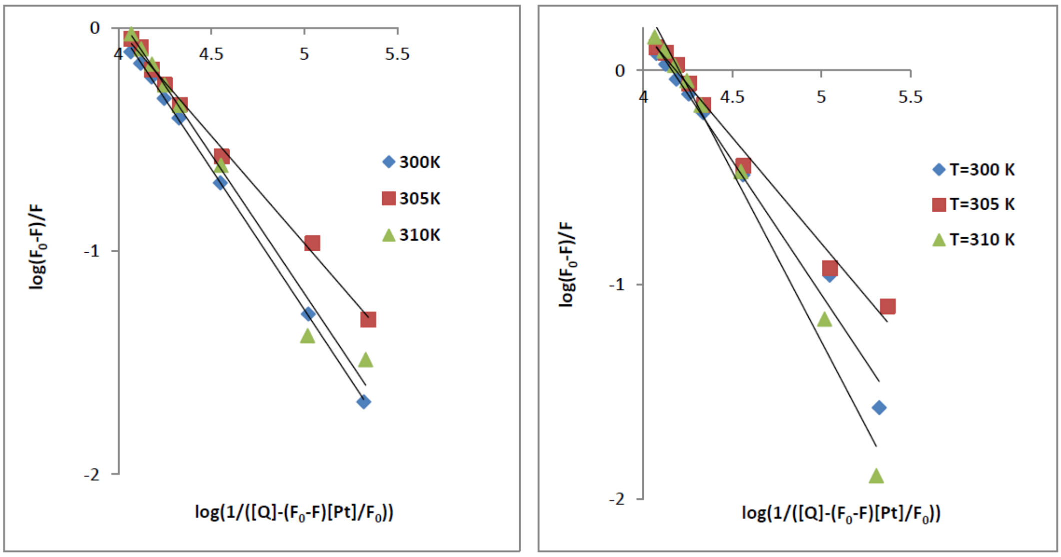

Figure S7. Plots of log(F0-F)/F versus log(1/([Q] − (F0-F)[Pt]/F0)) at different temperatures for HSA (10 μM, Ph = 7.40) in the presence of complexes **2b** (left) and **2c** (right).

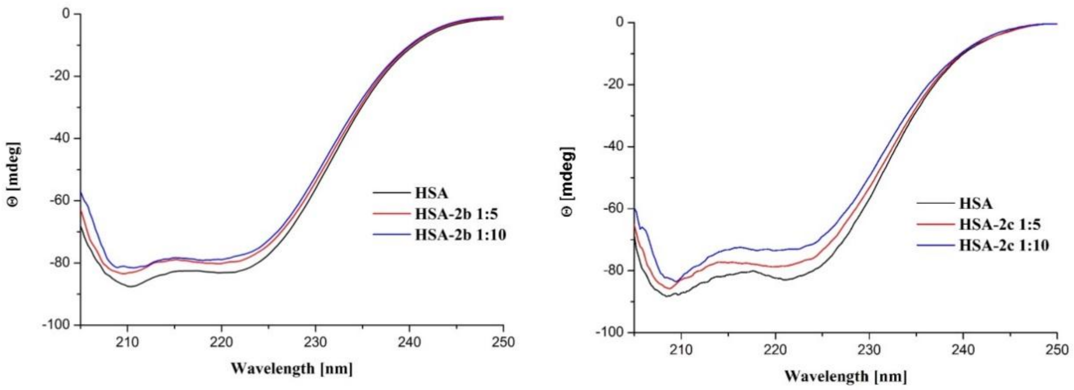

Figure S8. Circular dichroism spectra of HSA in the absence and presence of platinum complexes **2b** (left) and **2c** (right). [HSA] = 8 μM, pH = 7.40, 0.05M phosphate buffer, incubation at 37 °C during 24 h.

$$\frac{F_0}{F} = 1 + K_{SV}[Q] = 1 + K_q\tau_0[Q]\frac{F_0}{F} = 1 + K_{SV}[Q] = 1 + K_q\tau_0[Q] \quad (S1)$$

$$K_q = \frac{K_{SV}}{\tau_0} \quad (S2)$$

F_0 and F—fluorescence intensity in the absence and presence of a quencher, respectively;
K_{SV}—the Stern-Volmer constant;
[Q]—the concentration of quencher;
Kq—the quenching rate constant of protein;
τ_0—the lifetime of protein without the quencher (for HSA~5 ns).

$$\log\frac{F_0 - F}{F} = n\log K_A - n\log\left(\frac{1}{[Q_t] - (F_0 - F)[P_t]/F_0}\right) \quad (S3)$$

F_0 and F—fluorescence intensities in the absence and in the presence of the platinum complex, respectively;
$[Q_t]$ and $[P_t]$ are the total concentrations of the platinum complexes and HSA, respectively.

$$\ln K_q = -\frac{\Delta H^0}{RT} + \frac{\Delta S^0}{R} \quad (S4)$$

Kq—the bimolecular binding constant at the corresponding temperature (T);
R—the gas constant;
ΔH^0 and ΔS^0 are enthalpy and entropy change, respectively.

$$\Delta G^0 = \Delta H^0 - T\Delta S^0 \quad (S5)$$

ΔH^0—enthalpy change;
ΔS^0—entropy change;
ΔG^0—free energy change.
